# Attenuation of bile acid-mediated FXR and PXR activation in patients with Crohn’s disease

**DOI:** 10.1038/s41598-020-58644-w

**Published:** 2020-02-05

**Authors:** Aze Wilson, Ahmed Almousa, Wendy A. Teft, Richard B. Kim

**Affiliations:** 1 Clinical Pharmacology, Department of Medicine, Western University, 339 Windermere Rd, London, ON N6A 5A5 Canada; 2 Gastroenterology, Department of Medicine, Western University, 339 Windermere Rd, London, ON N6A 5A5 Canada; 3 Department of Physiology & Pharmacology, Western University, Medical Sciences Building, Rm 216, London, ON N6A 5C1 Canada

**Keywords:** Crohn's disease, Gastrointestinal models

## Abstract

Bile acids are endogenous ligands of nuclear receptors pregnane X (PXR) and farnesoid X (FXR). PXR and FXR regulate pathways that are impaired in inflammatory bowel disease (IBD). Decreases in PXR and FXR activity are documented in IBD; however reasons for this are unknown. We aimed to assess the effect of Crohn’s disease (CD) on the plasma bile acid composition *in vivo* and the resultant impact on PXR and FXR activation. A cross-sectional study evaluated the plasma concentrations of 12 bile acids in addition to 4β-hydroxycholesterol (4βOHC), an *in vivo* probe of the PXR target-gene cytochrome 3A4 (CYP3A4) and the FXR target-gene, fibroblast growth factor (FGF) 19 in individuals with (n = 74) and without (n = 71) CD. An *in vitro* model was used to assess the impact of CD-specific changes in the plasma bile acid composition on PXR and FXR activation. Decreases in glycochenodeoxycholic acid, taurocholic acid and lithocholic acid were seen in CD with increases in glycodeoxycholic acid and glycocholic acid relative to the total plasma bile acid profile. *In vitro*, increasing concentrations of bile acids applied in the same ratio as seen in the study cohorts resulted in decreased activation of both PXR and FXR in the CD model. *In vivo*, plasma 4βOHC (CD = 18.68 ng/ml ± 13.02 ng/ml, non-CD = 46.38 ng/ml ± 40.70 ng/ml, p ≤ 0.0001) and FGF19 (CD = 0.276 pg/L ± 0.189 pg/L, non-CD = 0.485 pg/L ± 0.42 pg/L, p = 0.0002) concentrations were lower in CD versus controls. Ultimately, CD-specific changes in the plasma bile acid composition lead to reduced activation of FXR and PXR target genes *in vitro* and *in vivo*.

## Introduction

Bile acids are a collection of dynamic “acidic steroids”, produced by the liver^1^. In health, physiologic concentrations of bile acids differ amongst the various compartments with the highest concentrations seen in the gallbladder (300 mmol/L) with decreasing concentrations in the hepatic canaliculi (20–50 mmol/L) and intestine (10 mmol/L)^2^. The role of bile acids *in vivo* extends beyond simple lipid absorption and metabolism and encompasses several key physiologic processes paramount to humans. These include regulation of xenobiotic exposure and drug metabolism, inflammatory pathways, and intestinal barrier function, mediated mainly through master regulators and transcription factors known as nuclear receptors^3–6^. Interestingly, synthesis and disposition of bile acids are tightly controlled by nuclear receptors, bile acid transporters, enzymes, and intestinal bacteria.

To date, more than 48 members of the nuclear receptor family have been identified^7^. Nuclear receptors, pregnane X (PXR) and farnesoid X (FXR) are important to drug metabolism and transport pathways *and* are increasingly recognized as being relevant to inflammatory bowel disease (IBD; Crohn’s disease, CD; ulcerative colitis, UC) pathogenesis^5,8–10^. Bile acids are important endogenous ligands of PXR and FXR, with varying degrees of potency^3,11^. PXR is most robustly activated by free or conjugated lithocholic acid (LCA) and deoxycholic acid (DCA), while FXR is most robustly activated by free or conjugated chenodeoxycholic acid (CDCA) and cholic acid (CA). FXR (gene *NR1H4*) is a sensor of intracellular bile acid concentrations within the liver and intestine and plays a key role in the regulation of the enterohepatic circulation^12,13^. Other target genes of FXR include key drug metabolism mediator, cytochrome P450 3A4 (*CYP3A4*), PXR (*NR1I2*), and the ileal hormone, fibroblast growth factor (FGF) 19 (*FGF19*)^14–16^. FXR also controls several genes that protect against intestinal inflammation, intestinal permeability and bacterial overgrowth^5,9,10^. PXR, also known as the steroid and xenobiotic-sensing nuclear receptor, plays an important role in the “detoxification” of the human body^3^. It is activated by a variety of endogenous and exogenous compounds, including bile acids. In turn, it heterodimerizes with the retinoid X receptor (RXR) to up-regulate the transcription of target genes that code for proteins necessary for the degradation and clearance of toxins from the body, including and importantly CYP3A4^3^. Additionally, i*n vivo* murine models of colitis have convincingly demonstrated PXR-mediated suppression of the nuclear factor kappa B (NFκB) inflammatory pathway^8^. Interestingly, unchecked NFκB activity, defects in the intestinal epithelial barrier and the excessive exposure of luminal bacteria and other antigens to the host lymphoid tissue are hallmarks of both CD and UC^17–19^. Limited data exploring the link between IBD pathogenesis and nuclear receptors suggest that PXR and FXR activity are repressed in IBD, providing a plausible mechanism for the dysregulation of such processes^20–23^. However, the reasons for reduced FXR and PXR activity in IBD are largely unknown.

To date, there is limited data describing the pattern of bile acids in IBD. Duboc *et al*. (2013) used liquid chromatography tandem mass spectrometry (LC-MS/MS) technology to evaluate plasma and fecal bile acids in subjects with UC and CD as well as healthy controls. They detected fundamental differences in fecal bile acids and linked these apparent disease-dependent changes to alterations in the gut microbial profile^24^. Similarly, Gnewuch *et al*. (2009) described differences in the ratio of primary to secondary bile acids using LC-MS/MS in subjects with UC versus non-IBD controls^25^. Accordingly, differences in bile acid synthesis or metabolism may be an underappreciated determinant of nuclear receptor signaling in IBD and may play a role in intestinal inflammation as alluded to by Pavlidis *et al*. (2015) in a comprehensive review of the topic^26^.

Consequently, we sought to assess the effect of CD presence and activity on the plasma bile acid profile *in vivo* and the resultant impact on the activation of PXR and FXR target genes *in vitro* and *in vivo*.

## Materials and Methods

### Subjects

A cross-sectional study was carried out in individuals with CD and non-CD healthy volunteers to evaluate cohort-specific plasma bile acid profiles as well as for the measurement of the endogenous biomarker for CYP3A4, 4βhydroxycholesterol (4βOHC) in plasma. The STROBE statement for cross-sectional studies was followed. Bile acid profiles were reconstructed in an *in vitro* model to evaluate PXR and FXR signaling.

Study groups were derived from CD patients being seen as part of the Personalized Medicine Program at Western University, London, Canada as well as individuals being seen for colorectal screening consultation or functional abdominal syndromes with previously normal colonoscopy between March 2013 and March 2017. Each subject provided two blood samples for analysis. Subjects from the CD arm had a histopathological diagnosis. CD subjects were excluded if there was missing information pertaining to their CD medical history. Individuals were included in the control arm if there was no history of cardiovascular, neurological, pulmonary, renal, hepatic, malignant or auto-immune disease. Controls were required to be never-smokers, to consume less than 5 alcoholic beverages weekly and to have had no prior history of surgical intervention pertaining to the cardiovascular, neurological, pulmonary, renal, hepatic, or intestinal systems. All subjects were required to be 18 years of age or older and to not be taking a concomitant CYP3A4 agonist or antagonist (macrolide antibiotics, ‘azole’ antifungals, nefazodone, verapamil, diltiazem, cyclosporine A, antiretrovirals, carbamazepine, phenytoin, lamotrigine, rifampin, rifabutin, dexamethasone, St. John’s Wort, bosentan). Additionally, subjects were excluded if there was a history of surgery (including intestinal resection or cholecystectomy) or if they had been exposed to antibiotics, probiotics, glucocorticoids or a bile salt sequestrant within the 4 weeks prior to study enrollment.

Data collected from the CD cohort included age, weight, sex, smoking history, CD phenotype, activity (assessed by the Harvey Bradshaw Index, HBI, where active disease was defined as a score of 4 or more), disease duration, medication use and hospitalizations^27^. Data collected from the control cohort included age, weight, sex, smoking history and alcohol consumption, medical and/or surgical history and any prescription or non-prescription drug use.

### Ethical considerations

Written, informed consent was obtained from each patient. The study received approval from the Western University Health Sciences Research Ethics Board (105930). All research was performed in accordance with relevant institutional regulations.

### Study objectives and outcomes

The objective of this study was to test the hypothesis that PXR and FXR activity is decreased in CD due to disease-dependent changes in the bile acid profile and that this has an inhibitory effect on downstream target genes. The primary endpoint was the differences in the bile acid profiles of the controls versus the CD population. Secondary outcomes included the mean plasma concentrations of CYP3A4 endogenous probe substance, 4βOHC and ileal hormone, FGF19 in a cohort of CD patients compared to non-CD controls.

Additionally, plasma bile acid profiles generated for each cohort (healthy volunteer, active CD, inactive CD) were incubated with each of two *in vitro* models of NR-signaling (PXR and FXR) to test the hypothesis that CD-specific differences in the bile acid profile induce differential FXR- and/or PXR-signaling, resulting in decreased downstream activation of its targets including CYP3A4 and the bile salt export protein (BSEP).

### Quantification of bile acid plasma concentrations

Plasma stored at −80^◦^C was used for quantification of 12 bile acids (cholic acid, CA; chenodeoxycholic acid, CDCA; deoxycholic acid, DCA; glycocholic acid, GCA; glycochenodeoxycholic acid, GCDCA; glycodeoxycholic acid, GDCA; lithocholic, LCA; taurocholic acid, TCA; taurodeoxycholic acid, TDCA; taurolithocholic acid, TLCA; tauroursodeoxycholic acid, TUDCA; and ursodeoxycholic acid, UDCA) using LC-MS/MS. Taurocholic acid-d5 (TCA-D5) was used as an internal standard (I.S). A standard curve of 1 nM to 40 µM was generated. Patient samples were homogenized by vortexing for 15 minutes at 4 °C @1400 RPM and incubated at −20 °C for 20 minutes. Samples were centrifuged at maximum speed at 4 °C for 30 minutes. Finally, 100 µL of supernatant was transferred to vials and 80 µL was injected into the LC-MS/MS system.

A two-dimensional LC system was used (Agilent HILIC plus column (4.6 * 50) 3.5 µm followed by Phenomenex 00B-4462-Y0 Kinetex 2.6 u C18 100 A (3.0 * 50)). A gradient elution technique was employed using the Agilent 1290 system. Mobile phase A consisted of 2 mM ammonium acetate (pH 4.15) with 10% acetonitrile. Mobile phase B consisted of acetonitrile: isopropyl-alcohol with water. The columns’ temperatures were maintained at 60 °C. Samples were eluted at a flow rate of 0.6 ml/min to 1.4 ml/min (0.1-12 minutes). Before injection of the subsequent sample, columns were cleaned with 100% mobile phase B for one minute and returned to a linear equilibrium for 7 minutes for a total run of 20 minutes for each sample. Mass detection occurred on a TSQ-Quantum Ultra mass spectrometer equipped with HESI source and operated in negative mode (4500 v spray voltage, 350 °C vaporizing temperature, 45 sheath gas pressure, 15 auxiliary gas pressure and 350 °C capillary temperature).

### *In vitro* luciferase transactivation assays

Human hepatocarcinoma (HepG2) cells, obtained from Cedarlane (Burlington, Ontario) were cultured in Dulbecco’s Modified Eagle Medium (VWR, Radnor, Pennsylvania) supplemented with 10% fetal bovine serum, L-glutamine, penicillin and streptomycin. Cell viability in the setting of bile acid exposure was assessed using the CellTiter-Glo Luminescent Cell Viability Assay purchased from Promega Corporation (Madison, Wisconsin) at serial concentrations and time points. Cells were then cultured in 12-well plates to 80% confluency in 3 days. Two HepG2 cell models were created by transient transfection using the lipofection method. In model 1, 0.5 µg of CYP3A4-XREM-pGL3, 0.5 µg of hPXR-pEF and 0.5 µg of renilla plasmid (pRL-CMV, Promega, Madison Wisconsin, USA) were co-transfected into HepG2 cells (PXR-CYP3A4 model). In model 2, 0.5 µg of bile salt export pump (BSEP)-pGL3, 0.5 µg of hFXR-pEF and 0.5 µg of pRL-CMV were co-transfected into HepG2 cells (FXR-BSEP model). Plasmids, CYP3A4-XREM-pGL3 and hPXR-pEF were previously prepared as described by Tirona *et al*.^28^ Plasmids, BSEP-pGL3 and hFXR-pEF were previously prepared as described by Marzolini *et al*.^29^. A corresponding “control” model was generated, with 0.5 µg of pGL3 basic (Promega, Madison Wisconsin, USA) and pEF (Invitrogen, Carlsbad California, USA) control vectors and 0.5 µg of pRL-CMV as a transfection a control. In model 1, PXR/CYP3A4-transfected cells were treated with one of the following: a mix of 12 bile acids (25 µM, 50 µM, or 75 µM) combined in a ratio representative of CD disease states (active CD, inactive CD) as well as that of a healthy individual; 0.1% DMSO in Optimem; or 10 µM of one of GDCA, DCA or LCA. In model 2, FXR/BSEP-transfected cells were treated with one of the following: a mix of 12 bile acids (25 µM, 50 µM, or 75 µM) combined in a ratio representative of CD disease states (active CD, inactive CD) as well as that of a healthy individual; 0.1% DMSO in Optimem; or 10 µM of one of CDCA, GCDCA or LCA. Cohort-specific bile acid profiles were derived from subject data presented in Supplementary Table 1. In both models, the 12-bile acid profile consisted of CA, TCA, GCA, CDCA, GCDCA, DCA, TDCA, GDCA, UCDCA, T-UCDCA, TLCA and LCA. All cells were incubated for 24 hours, washed with phosphate buffered saline and lysed with passive lysis buffer. Using the Promega Corporation Dual Luciferase Reporter Assay System (Madison, Wisconsin USA), luciferase activity was measured by Glomax 20/20 Luminometer (Promega, Madison Wisconsin, USA) and normalized by dividing the relative light units by the Renilla luciferase activity (firefly:renilla luciferase ratio, F/R ratio). The fold-change in luciferase activity was then estimated by dividing the F/R ratio for each cell group by the control (pGL3 basic or pEF control vector). Each experiment was performed in duplicate and repeated 3 times.

### Quantification of 4β-hydroxycholesterol plasma concentrations

Plasma concentrations of 4β-hydroxy-cholesterol (4β OHC), an endogenous *in vivo* probe of CYP3A4 activity, were determined by LC-MS/MS following sterol isolation by saponification and enhancement of the analyte product by picolinic acid derivatization as described by Honda *et al*. and Woolsey *et al*.^30,31^. A 4βOHC-standard curve from 0 to 200 ng/ml was created in Krebs-Henseleit Bicarbonate buffer. Fifty microlitres of subject plasma and 100 µl of standard were spiked with 1 µl of 1 mg/ml internal standard (d7-4βOHC) obtained from Avanti Polar Lipids (Alabaster, Alabama). Each aliquot was saponified in 500 µl of 1 M ethanolic potassium hydroxide at 37^◦^C for one hour followed by the addition of 150 µl of water. Sterols were twice extracted into hexane and further isolated by the evaporation of the hexane layers to dryness. Following this, 250 µl of a derivatization mixture (2-methyl-6-nitrobenzoic acid, 4-dimethylaminopyridine, picolinic acid, pyridine, triethylamine) was added to each sterol extract and incubated at 80^◦^C for one hour. Hexane (1 ml) was added to each aliquot and, following centrifugation at 14,000 rpm, the supernatant was collected and evaporated to dryness at 80^◦^C. Following re-constitution in acetonitrile and sodium chloride, a 20µl-aliquot was injected into the LC-MS/MS system. An Agilent C18 Zorbax Eclipse Plus column (100 × 2.1 mm, 1.8 µm) was used with mobile phases of 0.1% formic acid in water and 50:50 acetonitrile in methanol with 0.1% formic acid. The retention times for d7-4βOHC and 4βOHC were 8 minutes and 8.1 minutes respectively with each analyte detected in positive mode (mass transitions of 642.4 > 146.5 m/z, d7-4βOHC and 635.4 > 146.5 m/z, 4βOHC). The lower limit of detection of plasma 4βOHC was 2.5 ng/ml.

### Quantification of fibroblast growth factor 19 plasma concentrations

Plasma concentrations of FGF19 were determined by a commercial enzyme-linked immunosorbent assay (ELISA) kit (FGF19 Quantikine ELISA kit, category no. DF1900; R&D Systems, Minneapolis, MN, US). A colorimetric method for detection and estimation of FGF19 plasma concentrations was used following the manufacturer’s instructions. All aliquots as well as the standard curve (0 pg/ml-1000pg/ml) were assayed in duplicate.

### Statistical analyses

The sample size calculation was based on the secondary objective. A mean 4βOHC plasma concentration of 30 ng/mL ± 15 ng/mL has been reported in healthy controls^32^ We calculated that a minimum of 140 subjects (70 per group) would need to be enrolled in the study to have a statistical power of 80% to detect a minimum 23%-difference between the CD and control cohorts with a two-sided significance level of 0.05.

All statistical analyses were carried out in Graphpad Prism version 5 and SPSS version 17. Mean plasma concentrations for the 12 aforementioned bile acids were compared between the control group and the CD group (sub-divided by disease activity) using a Student’s t-test. A one-way ANOVA with Student’s *t*-test and Bonferroni post hoc test were used to compare 4βOHC and FGF19 plasma concentrations between CD (active, inactive disease, n = 74) and control groups (n = 71). A p-value < 0.05 was considered significant. A multiple linear regression analysis was used to further evaluate the relationship between presence or absence of CD, other covariates and the inter-individual variation in 4βOHC and FGF19 plasma concentrations (natural log-transformed) respectively. Other covariates assessed included the following: sex, age, weight, and disease activity. A p-value ≤ 0.05 was considered significant.

Similarly, for the *in vitro* experiments, a one-way ANOVA with Tukey’s multiple comparisons test identified any significant differences between cell groups. A p-value < 0.05 was considered significant.

## Results

**Figure 1:**
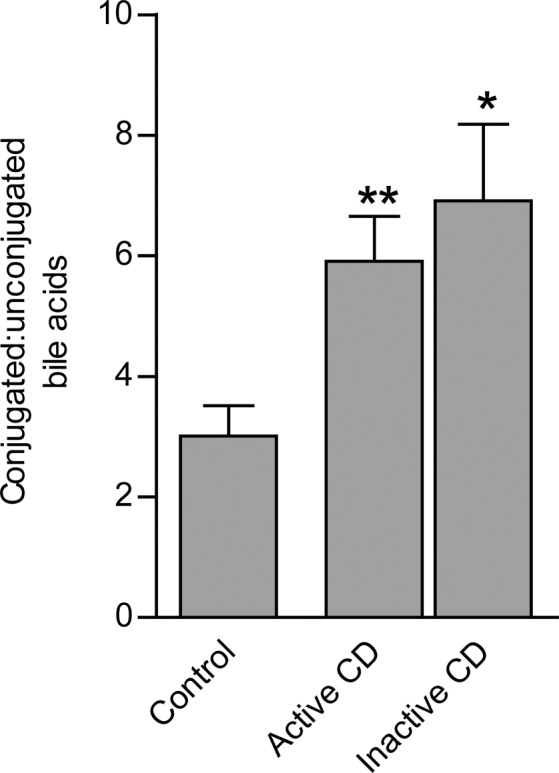
The ratio of the plasma concentration of conjugated to unconjugated bile acids stratified by cohort (control, active CD, inactive CD). The 95%CI is represented by the vertical T-line. *p < 0.05; **p < 0.01; ***p < 0.001. Crohn’s disease, CD.

Supplementary Figure 1 summarizes patient study flow. Table 1 summarizes the demographic data as well as the clinical parameters for both populations. Plasma bile acid profiles consisting of 12 bile acids were determined for all subjects (n = 145) and stratified by disease presence and activity (Supplementary Figure 2). Table 2 summarizes the plasma bile acid profiles as a percentage of the total bile acid profile. Data is presentation as mean concentrations in Supplementary Table 1. Subjects with CD had a higher ratio of conjugated to unconjugated bile acids compared to controls (active CD = 5.9; inactive CD = 6.9; control = 3.0, Fig. 1). Significant differences in the composition of the bile acid profile were seen based on presence or absence of CD. Differences in the percent composition of GCDCA, GCA, TCA, GDCA, and LCA were most significant (Table 2). CDCA and DCA were different between controls and CD subjects with active disease. There was no difference in UDCA, TDCA, CA.

**Table 1:** Demographics.

Variables	Crohn’s disease (n = 74)	Control population (n = 71)
Age, years (mean, range)	38.46 (18-72)	45.3 (19-71)
Female sex (%)	43 (58.1)	43 (60.6)
Weight, kg (mean ± std)	79.25 ± 17.88	81.65 ± 19.33
CD location		
Ileal (%)	32 (43.2)	—
Colonic (%)	6 (8.1)	—
Ileo-colonic (%)	36 (48.6)	—
Disease duration, years (mean ± std)	6.79 ± 7.90	—
Active disease, HBI > 4 (%)	30 (40.5)	
Smoking history (%)	17 (23.0)	0 (0)
Current medications		0 (0)
5-ASA (%)	0 (0.0%)	—
MTX (%)	10 (8.6%)	—
Thiopurine (%)	62 (53.4%)	—
Anti-TNF (%)	64 (55.2%)	—
Combination therapy (%)	42 (36.2%)	—
Surgery (%)	0 (0)	0 (0)
Hospitalizations (mean ± std)	0.41 ± 0.93	—

Kilograms, kg; standard deviation, std; inflammatory bowel disease, IBD; Crohn’s disease, CD; Harvey Bradshaw Index, HBI; tumor necrosis factor, TNF; 5-aminosalicylate, 5-ASA; methotrexate, MTX.

**Table 2:** Bile acid profiles presented as a percentage of the total plasma bile acid profile by group.

	Plasma Bile acids	Control (n = 71)	Inactive CD (n = 44)	Active CD (n = 30)	p-value*
Primary Bile acids plus conjugates	CDCA	4.87%	6.75%	8.76%	ns
	G-CDCA	47.11%	33.11%	37.02%	**<0.001**
	**Total**	**51.98%**	**39.86%**	**45.78%**	
	CA	4.87%	2.61%	2.84%	ns
	G-CA	1.08%	4.79%	4.98%	**<0.001**
	T-CA	3.01%	1.31%	0.90%	**<0.01**
	**Total**	**8.96%**	**8.71%**	**8.72%**	
Secondary bile acids plus conjugates	DCA	5.44%	5.74%	3.19%	ns
	G-DCA	14.46%	32.00%	31.32%	**<0.001**
	T-DCA	3.20%	3.97%	2.19%	Ns
	**Total**	**23.10%**	**41.71%**	**36.70%**	
	LCA	6.49%	3.50%	1.46%	**<0.001**
	T-LCA	1.33%	0.09%	0.38%	ns
	**Total**	**7.82%**	**3.59%**	**1.84%**	
Tertiary bile acids plus conjugates	UDCA	6.97%	6.17%	6.91%	ns
	T-UDCA	0.08%	0.02%	0.03%	ns
	**Total**	**7.05%**	**6.19%**	**6.94%**	

*Comparison of control to active and inactive CD. Crohn’s disease, CD; cholic acid, CA; chenodeoxycholic acid, CDCA; deoxycholic acid, DCA; glycocholic acid, GCA; glycochenodeoxycholic acid, GCDCA; glycodeoxycholic acid, GDCA; lithocholic acid, LCA; taurocholic acid, TCA; taurodeoxycholic acid, TDCA; taurolithicholic acid, TLCA; tauroursodeoxycholic acid, TUDCA; and ursodeoxycholic acid, UDCA; not significant, ns.

To explore the impact of CD-dependent alterations in the plasma bile acid profile on PXR and FXR activation, transient transfection cell models and luciferase transactivation assays were used. HepG2 viability was assessed using a luminescent cell viability assay following exposure to various individual bile acids or a combined mix of 12 bile acids, in proportions seen among those with CD and control subjects (Table 2, Supplementary Figure 2). With the exception of exposure to LCA concentrations >10 µM, HepG2 cells remained viable during the bile acid incubation period.

**Figure 2:**
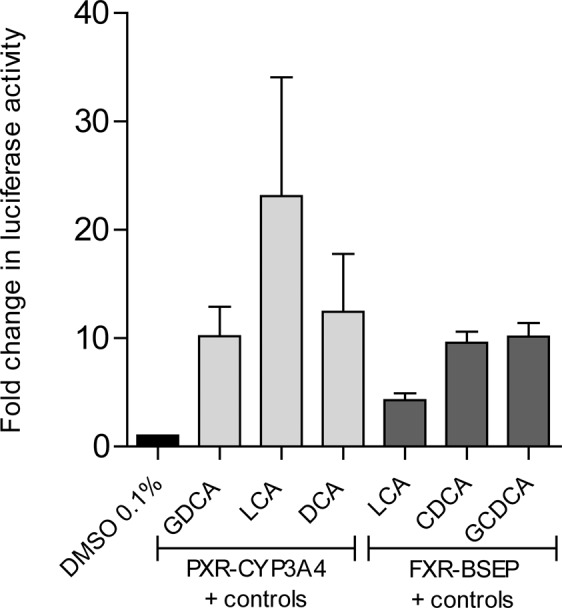
Effect of known bile acid agonists on CYP3A4-pGL3 basic reporter (light grey bars) or BSEP-pGL3 basic reporter (dark grey bars) activity in HepG2 cells co-transfected with *hPXR* or *hFXR* respectively or a vector control. In addition to 0.1%DMSO, one of DCA, LCA, or GDCA (10 µM) were incubated with cells for 24 hours as positive controls to validate the PXR-CYP3A4. Similarly, one of GCDCA, LCA or CDCA (10 µM) was incubated with cells for 24 hours as positive controls to validate the FXR-BSEP. Data are presented relative to the vector control. Data are presented as mean ± SEM. Three independent experiments were performed for each model. Deoxycholic acid, DCA; lithocholic acid, LCA; glycodeoxycholic acid, GDCA; glycochenodeoxycholic acid, GCDCA; lithocholic acid, LCA; chenodeoxycholic acid, CDCA; Crohn’s disease, CD; dimethyl sulfoxide, DMSO; pregnane X receptor, PXR; farnesoid X receptor, FXR; cytochrome p450, CYP; bile salt export pump, BSEP; pGL3, luciferase; hepatocarcinoma, Hep; human, h; standard error of the mean, SEM.

**Figure 3:**
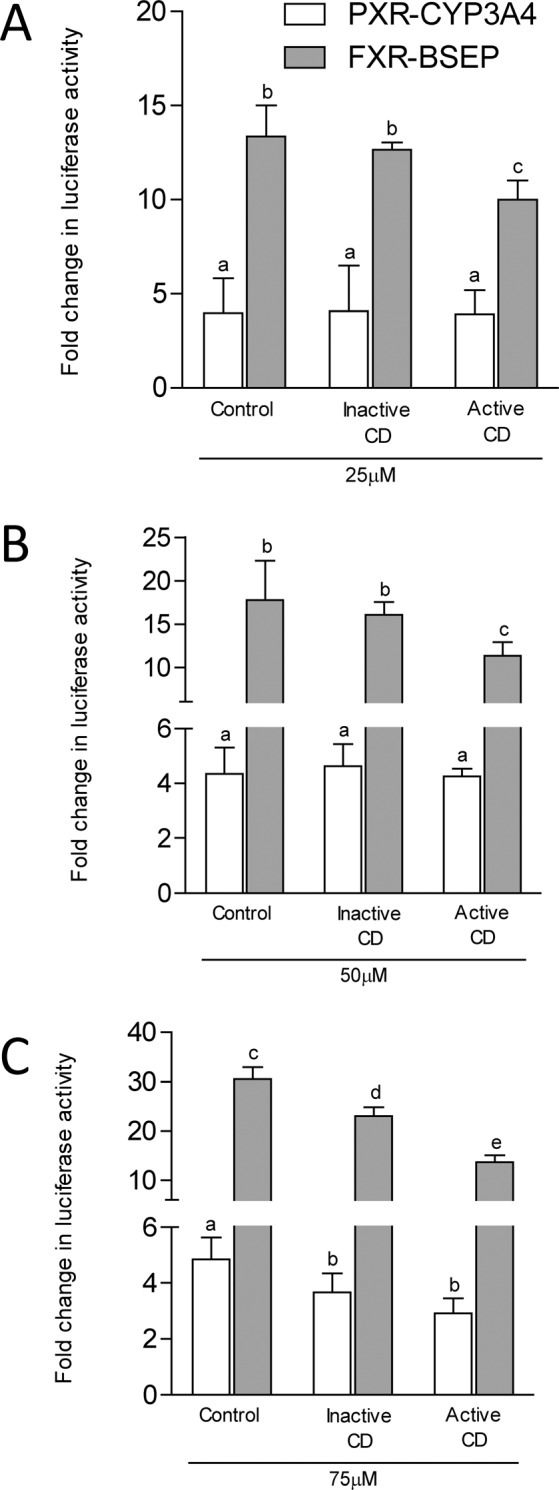
Effect of patient-derived plasma bile acid profiles on CYP3A4-pGL3 basic reporter (white bars) or BSEP-pGL3 (grey bars) basic reporter activity in HepG2 cells co-transfected with *hPXR* or *hFXR* respectively or a vector control. Bile acid profiles in increasing concentrations (Panel A, 25 µM; Panel B, 50 µM; Panel C, 75 µM) representative of inactive or active CD or a healthy population were incubated with cells for 24 hours. Data are presented relative to the vector control. Data are presented as mean ± SEM. Different letters represent statistically significant differences among groups. Three independent experiments were performed for each model. Crohn’s disease, CD; pregnane X receptor, PXR; cytochrome p450, CYP; farnesoid X receptor, FXR; bile salt export pump, BSEP; pGL3, luciferase; hepatocarcinoma, Hep; human, h; standard error of the mean, SEM.

PXR-mediated transactivation of CYP3A4 promoter construct, measured using luciferase activity, demonstrated markedly elevated activity in the presence of DCA (12-fold), GDCA (10-fold) or LCA (23-fold) (Fig. 2). These data are in agreement with other reports of bile acid-induced activation of PXR^33,34^ Conversely, no difference was seen in the luciferase activity amongst the transfected cells exposed to the cohort-specific bile acid profiles at total concentration 25 µM (Fig. 3A) or 50 µM (Fig. 3B). At 75 µM, bile acid-activated reporter activities were significantly decreased in the CD cohorts compared to the control population, although no difference was seen between active and inactive CD (Fig. 3C). When the BSEP promoter construct known to be transactivated by FXR was transfected into HepG2 cells, luciferase activity was markedly increased in the presence of known FXR agonists CDCA and GCDCA, and while a more modest activation was noted for LCA (Fig. 2)^11^ At a total bile acid concentration of 25 µM (Fig. 3A) and 50 µM (Fig. 3B), reduced FXR-mediated activation of BSEP was seen with the active CD bile acid profile compared to the healthy volunteer profile. At 75 µM (Fig. 3C), bile acid-activated reporter activities were significantly decreased in the disease state and further so in active disease.

Additionally, seventy-four subjects with CD and 71 controls were included to evaluate PXR and FXR target gene activation. PXR activation was assessed using the *in vivo* CYP3A4 activity using the endogenous probe, 4βOHC in plasma while FXR activation was assessed using FGF19 plasma concentrations.

**Figure 4:**
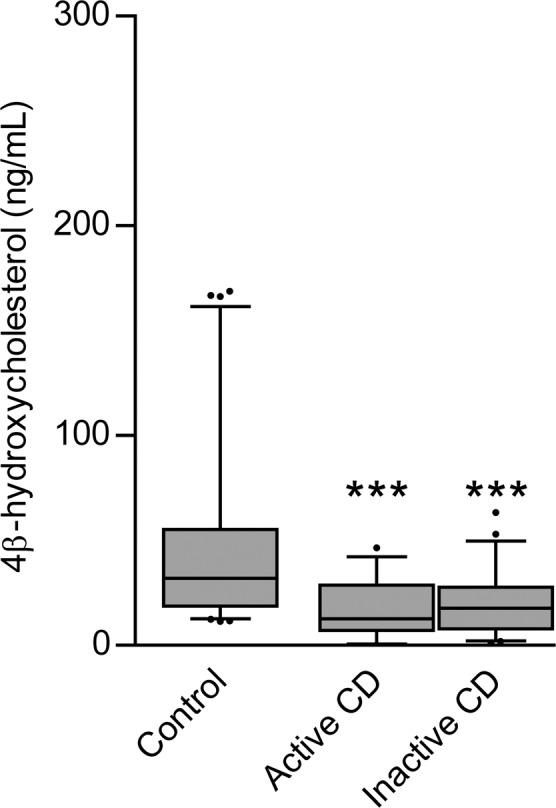
The mean 4βOHC plasma concentrations for subjects with active and inactive Crohn’s disease (CD) as well as non-CD controls. Median values (thick horizontal line), 25th and 75th percentile values (box outline), minimum and maximum values (whiskers), and outlier values (circles). *p < 0.05; **p < 0.01; ***p < 0.001. 4β-hydroxycholesterol, 4βOHC.

The 4βOHC plasma concentrations were significantly lower in the total CD population compared to the non-CD controls (CD = 18.68 ng/ml ± 13.02 ng/ml, non-CD = 46.38 ng/ml ± 40.70 ng/ml, p ≤ 0.0001) (Fig. 4) on bivariate analysis and when adjusting for the covariates, age, sex, weight and disease activity (Supplementary Table 2). The multiple linear regression analysis accounted for 33.6% of the inter-individual variation in 4βOHC plasma concentrations. No significant difference was seen in 4βOHC plasma concentrations between CD subjects stratified by disease activity (inactive CD = 19.75 ng/ml ± 13.34 ng/ml, active CD = 17.11 ng/ml ± 12.60 ng/ml, p = 0.99) (Fig. 4).

**Figure 5:**
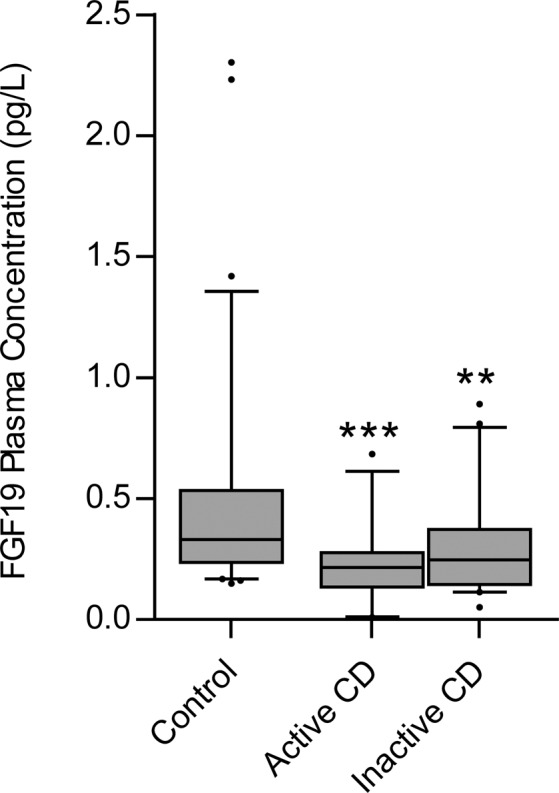
The mean FGF19 plasma concentrations for subjects with active and inactive Crohn’s disease (CD) as well as non-CD controls. Median values (thick horizontal line), 25th and 75th percentile values (box outline), minimum and maximum values (whiskers), and outlier values (circles). *p < 0.05; **p < 0.01; ***p < 0.001. Fibroblast growth factor 19, FGF19.

The FGF19 plasma concentrations were significantly lower in the total CD population compared to the non-CD controls (CD = 0.276 pg/L ± 0.189 pg/L, non-CD = 0.485 pg/L ± 0.42 pg/L, p = 0.0002) (Fig. 5) on bivariate analysis and when adjusting for the covariates, age, sex, weight and disease activity (Supplementary Table 3). The multiple linear regression analysis accounted for 15% of the inter-individual variation inFGF19 plasma concentrations. No significant difference was seen in FGF19 plasma concentrations between CD subjects stratified by disease activity (inactive CD = 0.305 pg/L ± 0.032 pg/L, active CD = 0.237 pg/L ± 0.029 pg/L, p = 0.12) (Fig. 5).

## Discussion

Nuclear receptors as mediators of inflammatory bowel disease pathogenesis as well potential therapeutic targets for the treatment of such conditions are areas of active investigation and drug development. PXR and FXR are of particular relevance to human disease and xenobiotic response due to their role in the detoxification of endo/xenobiotics and regulation of the expression and activity of clinically important hepatic and intestinal enzymes and transporters^35^. *In vitro* and *in vivo* models have demonstrated a decrease in PXR and FXR signaling in IBD, with a concomitant improvement in intestinal inflammatory lesions with PXR and FXR agonism^9,10,20,36^. Interestingly, knocking out *PXR* or *FXR* in murine models does not induce a spontaneous colitis; however, it facilitates the onset and increases the severity of the inflammatory insult, suggesting that these nuclear receptors may contribute to, but are not wholly responsible for, IBD pathogenesis^9^.

We tested the hypothesis that differences in the IBD-associated plasma bile acid profile composition in patients with active as well as inactive CD, relative to healthy subjects result in differential PXR and FXR activation. We showed that shared FXR-PXR target, CYP3A4 is down-regulated in a population of individuals with CD compared to non-CD controls.

Bile acid data are presented as percentages relative to the total bile acids detected for each cohort rather than absolute quantities. Given this, we observed differences in the plasma bile acid profiles of CD and non-CD populations. Specifically, a difference in the percent composition of potent PXR ligand, LCA was noted between groups. Additionally, an increased percentage of GDCA and GCA and a decreased percentage of TCA and GCDCA were seen among those with CD (Table 2; Supplementary Figure 2). It is possible that the observed differences may be due to documented changes in the presence of bile acid-modifying gut bacteria^24,37^. A limitation of this study is that we did not evaluate the bile acid composition in other compartments *in vivo* (liver, intestine, bile, feces). In a study by Swann *et al*.^38^, authors found that bile acid concentration profiles were tissue-specific; differences in conjugation were seen between, heart, liver and kidney bile profiles generated in a rat model^38^ Additionally, Hofmann reported the highest bile acid concentrations are present in the gall bladder (300 mmol/L) and hepatic canaliculi (20–50 mmol/L) with lower concentrations within the intestine (10 mmol/L)^2^.

Importantly, we showed that disease-dependent differences in bile acid profiles may account for differential FXR and PXR signaling in CD. HepG2 cell were transfected with one of the following: FXR and a BSEP-luciferase promoter construct, which our group had previously demonstrated to be transactivated by FXR, *or* PXR and a CYP3A4-luciferase promoter construct transactivated by PXR^28,39^ When we incubated our *in vitro* models with a mixture of bile acids in the same percent composition seen in CD patients relative to the control subjects, a clear difference in bile acid-induced activation of the nuclear receptors between control and diseased cohorts was noted. The total bile acid concentrations used to assess PXR or FXR activity *in vitro* were selected based on the fact that observed plasma bile acid concentrations are quite low, compared to measured bile and liver concentrations which can readily exceed 100-fold greater than plasma concentration^2,40^. Our findings clearly suggest that bile acid-mediated PXR activation is reduced in those with CD, due to lower circulating concentration of high affinity bile acid PXR agonists such as LCA.

To address the *in vivo* impact of PXR and FXR expression in patients with CD, we measured the presence of 4β OHC, an endogenous marker/probe of CYP3A4 (a shared PXR-FXR substrate) as well as the ileal hormone, FGF19 in our populations^32^. Indeed, even when we took into account the effect age, sex, weight and disease activity (assessed by HBI) there was clear reduction in measured 4β OHC in CD patients, suggesting reduced CYP3A4 expression in patients with CD (Supplementary Table 2).

Similarly, plasma FGF19 was significantly reduced in our CD population compared to controls, suggesting diminished FXR activation in our CD population. The majority of the individuals with CD had ileal involvement with or without colonic involvement (Table 1); however, the extent of ileal involvement was not known. This may introduce an element of confounding into our dataset and the results should be interpreted with caution.

## Conclusion

Bile acid sensing nuclear receptors PXR and FXR are increasingly recognized as key modulators of inflammatory bowel disease in humans. We now demonstrate that patients with CD, due to differences in their bile acid profile, do not activate PXR and FXR to the extent seen in those *without* CD, and thereby exhibit reduced CYP3A4 activity and FGF19 expression. Given the central role of CYP3A4 to the metabolism of over 50% of drugs in clinical use, including the corticosteroids prednisone and budesonide, as well as newer targeted agents such as tofacitinib used to treat IBD, our findings add important new insights on the interplay between bile acid composition in CD and nuclear receptors of direct relevance to optimal dosing of CYP3A4 substrate drugs in patients with CD.

## Supplementary information

Supplementary Tables and Figures.
