# Supplementary material for: Attenuation of bile acid-mediated FXR and PXR activation in patients with Crohn’s disease
Source: Sci Rep. 2020 Feb 5;10:1866. doi: 10.1038/s41598-020-58644-w (PMC7002620; doi:10.1038/s41598-020-58644-w)
Supplement: Supplementary file 1 — Supplementary Tables and Figures. [file 41598_2020_58644_MOESM1_ESM.pdf]

Supplementary Material

Attenuation of bile acid-mediated FXR and PXR activation in patients with Crohn's disease

Aze Wilson, Ahmed Almousa, Wendy A. Teft, Richard B. Kim

Supplementary Table 1: Bile acid profiles presented as mean concentrations with standard deviations

Supplementary Table 2: Multiple linear regression model for the effect on Ln-transformed 4β-hydroxycholesterol plasma concentrations for the total population

Supplementary Table 3: Multiple linear regression model for the effect on Ln-transformed FGF19 plasma concentrations for the total population

Supplementary Figure 1. Study subject selection and procedure pathway.

Supplementary Figure 2. Bile acid compositions in plasma.

Supplementary Table 1: Bile acid profiles presented as mean concentrations with standard deviations (ng/mL)

Bile acid (ng/mL)	Control (n=71)	Inactive CD (n=44)	Active CD (n=30)	p-value*
CA	200.6±777.1	176.6±419.7	184.6±343.0	ns
TCA	47.8±31.0	43.9±37.8	44.7±44.2	**<0.01**
GCA	71.5±134.1	276.3±297.9	256.5±224.2	**<0.001**
CDCA	342.8±1840.3	519.3±1321.4	472.3±512.4	ns
GCDCA	1705.3±1992.6	1984±1674	2730±2929	**<0.001**
DCA	202.2±563.8	368.3±997.9	274.9±400.1	ns
TDCA	106.8±227.5	253.8±550.3	176.8±238.1	ns
GDCA	473.2±592.1	2523±2783	3587±6810	**<0.001**
LCA	96.5±67.3	114.6±109.8	86.8±66.1	**<0.001**
TLCA	11.1±56.0	8.3±22.3	17.5±32.5	ns
UDCA	175.8±244.3	331.4±412.6	434.7±422.9	ns
TUDCA	1.7±5.8	0.7487±4.6	0.8586±4.7	ns

*Comparison of control to active and inactive CD

Crohn's disease, CD; cholic acid, CA; chenodeoxycholic acid, CDCA; deoxycholic acid, DCA; glycocholic acid, GCA; glycochenodeoxycholic acid, GCDCA; glycodeoxycholic acid, GDCA; lithocholic acid, LCA; taurocholic acid, TCA; taurodeoxycholic acid, TDCA; taurolithicholic acid, TLCA; tauroursodeoxycholic acid, TUDCA; and ursodeoxycholic acid, UDCA; not significant, ns.

Supplementary Table 2: Multiple linear regression model for the effect on Ln-transformed 4β-hydroxycholesterol plasma concentrations for the total population (n = 145)

Variable	β-coefficients	Standard error	P-value
**Intercept**	2.721	0.447	**≤0.0001**
**Sex**	0.548	0.151	**≤0.0001**
**Age**	0.130	0.005	**0.008**
**Weight**	0.000	0.004	0.92
**Crohn's disease**	-0.832	0.162	**≤0.0001**
**Disease activity (HBI>4)**	-0.200	0.197	0.31

Harvey Bradshaw Index, HBI

Supplementary Table 3: Multiple linear regression model for the effect on Ln-transformed FGF19 plasma concentrations for the total population (n = 145)

Variable	β-coefficients	Standard error	P-value
**Intercept**	-0.765	-0.383	**0.048**
**Sex**	0.123	0.137	0.37
**Age**	0.001	0.004	0.73
**Weight**	-0.004	0.004	0.28
**Crohn's disease**	-0.349	0.180	**0.02**
**Disease activity (HBI>4)**	-0.503	0.149	**0.006**

Harvey Bradshaw Index, HBI; fibroblast growth factor 19, FGF19

Supplementary Figure 1. Study subject selection and procedure pathway.

Inflammatory bowel disease, IBD; Crohn's disease, CD; 4β-hydroxycholesterol, 4βOHC; fibroblast growth factor 19, FGF19.

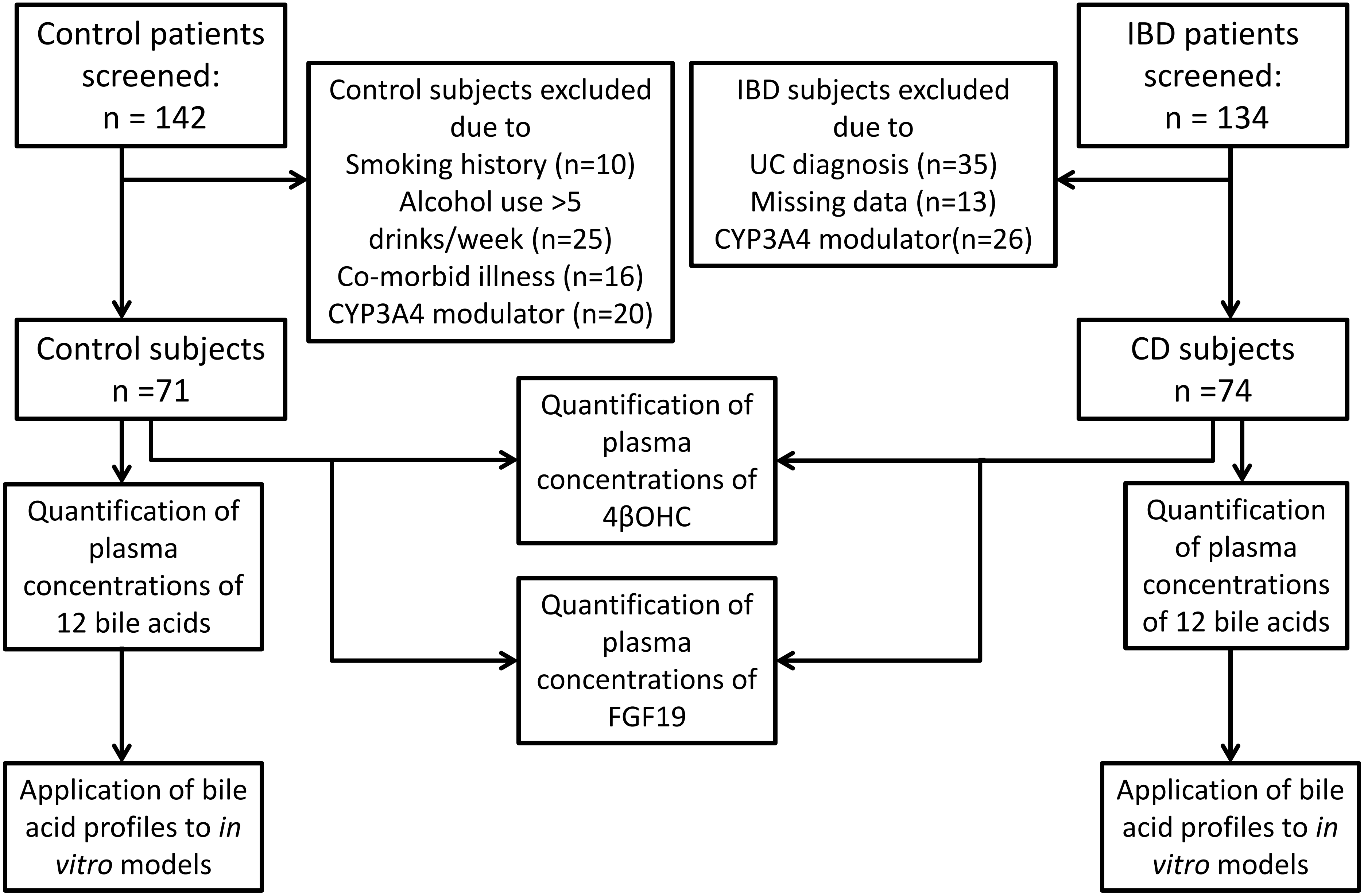

Control patients screened: n = 142
Control subjects excluded due to
Smoking history (n=10)
Alcohol use >5 drinks/week (n=25)
Co-morbid illness (n=16)
CYP3A4 modulator (n=20)
IBD subjects excluded due to
UC diagnosis (n=35)
Missing data (n=13)
CYP3A4 modulator(n=26)
IBD patients screened: n = 134
Control subjects n =71
CD subjects n =74
Quantification of plasma concentrations of 4βOHC
Quantification of plasma concentrations of FGF19
Quantification of plasma concentrations of 12 bile acids
Quantification of plasma concentrations of 12 bile acids
Application of bile acid profiles to in vitro models
Application of bile acid profiles to in vitro models

Supplementary Figure 2. Bile acid compositions in plasma.

The mean individual plasma bile acid profiles expressed as a percentage of the total plasma bile acid profile, stratified by cohort (control, active CD, inactive CD). Crohn's disease, CD; cholic acid, CA; chenodeoxycholic acid, CDCA; deoxycholic acid, DCA; glycocholic acid, GCA; glycochenodeoxycholic acid, GCDCA; glycodeoxycholic acid, GDCA; lithocholic acid, LCA; taurocholic acid, TCA; taurodeoxycholic acid, TDCA; taurolithocholic acid, TLCA; tauroursodeoxycholic acid, TUDCA; and ursodeoxycholic acid, UDCA.

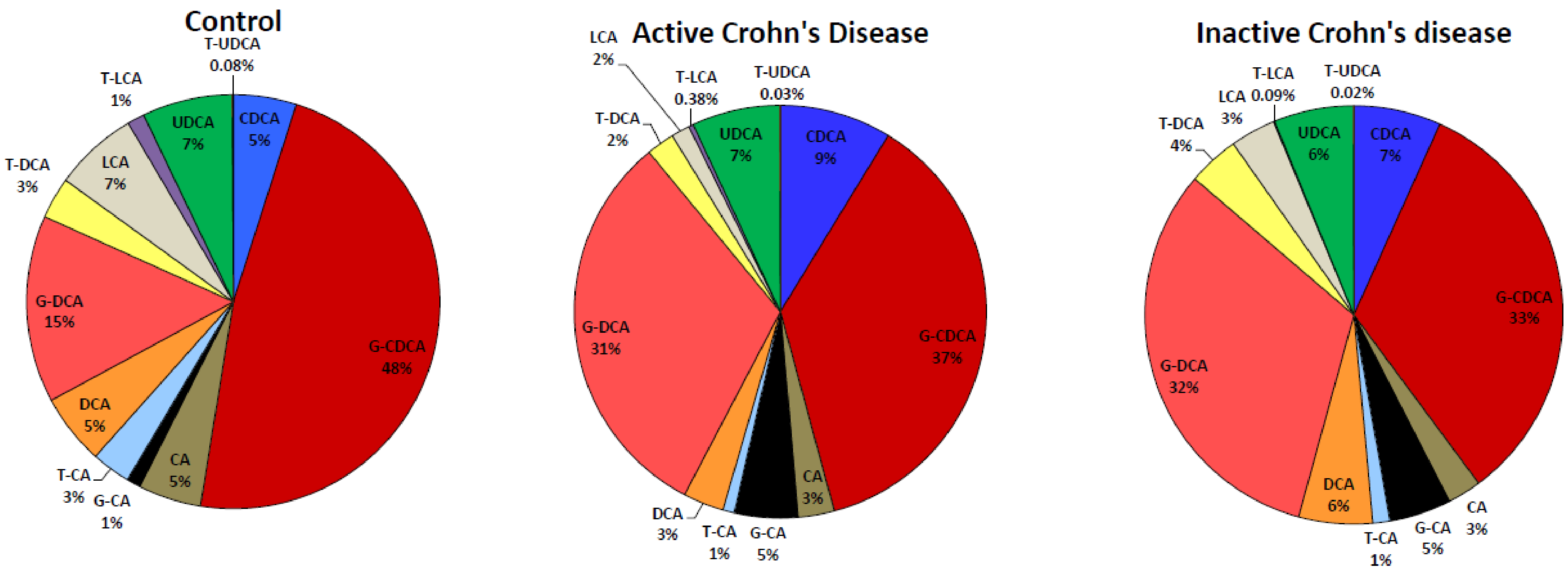

Control
T-UDCA 0.08%
CDCA 5%
G-CDCA 48%
CA 5%
G-CA 1%
T-CA 3%
DCA 5%
G-DCA 15%
T-DCA 3%
LCA 7%
T-LCA 1%
UDCA 7%
Active Crohn's Disease
T-UDCA 0.03%
CDCA 9%
G-CDCA 37%
CA 3%
G-CA 5%
T-CA 1%
DCA 3%
G-DCA 31%
T-DCA 2%
LCA 2%
T-LCA 0.38%
UDCA 7%
Inactive Crohn's disease
T-UDCA 0.02%
CDCA 7%
G-CDCA 33%
CA 3%
G-CA 5%
T-CA 1%
DCA 6%
G-DCA 32%
T-DCA 4%
LCA 3%
T-LCA 0.09%
UDCA 6%
